# Motor corticospinal excitability: a novel facet of pain modulation?

**DOI:** 10.1097/PR9.0000000000000725

**Published:** 2019-03-08

**Authors:** Yelena Granovsky, Elliot Sprecher, Alon Sinai

**Affiliations:** a The Laboratory of Clinical Neurophysiology, Technion Faculty of Medicine, Haifa, Israel; b Department of Neurology, Rambam Health Care Campus, Haifa, Israel; c Department of Neurosurgery, Rambam Health Care Campus, Haifa, Israel

**Keywords:** Pain modulation, Conditioned pain modulation, Temporal summation, Motor cortex, Corticospinal excitability, Motor-evoked potentials, Transcranial magnetic stimulation

## Abstract

**Introduction:**

Increase in excitability of the primary motor cortex (M1) is associated with pain inhibition by analgesics, which is, in turn, associated with the psychophysical antinociceptive pain modulation profile. However, the relationship between neurophysiological M1 excitability and psychophysical pain modulation has not yet been explored.

**Objectives:**

We aim to study these relationships in healthy subjects.

**Methods:**

Forty-one young healthy subjects (22 women) underwent a wide battery of psychophysical testing that included conditioned pain modulation (CPM) and pain temporal summation, and a transcranial magnetic stimulation neurophysiological assessment of the motor corticospinal excitability, including resting motor threshold, motor-evoked potentials (MEPs), and cortical silent period.

**Results:**

Increased motor corticospinal excitability in 2 parameters was associated with more efficient CPM: (1) higher MEP amplitude (*r* = −0.574; *P*__Bonferroni_ = 0.02) and (2) longer MEP duration (*r* = −0.543; *P*__Bonferroni_ = 0.02). The latter also correlated with the lower temporal summation magnitude (*r* = −0.421; *P* = 0.007); however, on multiplicity adjustment, significance was lost.

**Conclusions:**

Increased corticospinal excitability of the primary motor cortex is associated with more efficient inhibitory pain modulation as assessed by CPM, in healthy subjects. Motor-evoked potential amplitude and duration may be considered as an additional, objective and easy to measure parameter to allow for better individual assessment of pain modulation profile.

## 1. Introduction

Higher excitability of the primary motor cortex and the efficient inhibitory facet of endogenous pain modulation are associated with analgesic responses and can therefore be considered as neurophysiological and psychophysical correlates of antinociception. Indeed, from the neurophysiological point of view, the stimulation of primary motor cortex (M1) through high-frequency repeated transcranial magnetic stimulation (rTMS) or electrical stimuli, as well as its activation through physical exercises, exerts inhibitory effects on the pain system through activation of limbic, cortical, and subcortical brain areas associated with antinociception.^22,43,53^ From psychophysical point of view, the activation of the same brain structures can be observed during induction of endogenous analgesia using several experimental methods (ie, offset analgesia^37^; stress-induced analgesia^80^; and conditioned pain modulation [CPM] response).^6,50,55,63^ Furthermore, similar to stimulation-induced M1 activation, various pain-alleviating treatments increase the efficiency of descending pain inhibition, as was reported for CPM.^33,51,67,76,77^

Complementary to psychophysical assessment of endogenous pain inhibition, activation and measuring of spatial and temporal summation (TS) of pain can be considered psychophysical methods for evaluation of ascending pain facilitatory pathways. In comparison with simple pain threshold and suprathreshold pain estimation, where the contribution of peripheral components can be considered, the mechanisms of pain summation are centrally mediated, reflecting central neuronal sensitization state.^4,16,73^ The assessment of TS is widely used in clinical studies; enhanced TS magnitude characterizes many chronic pain states^3,45,65,69^ and was explored as a predictor of acute postoperative pain.^71^ Therefore, the combination of less-efficient pain inhibition and enhanced pain facilitation presumably characterizes a pronociceptive state of pain modulation.^24,78^ We addressed such inhibition and facilitation using CPM and TS, which are most widely explored for the assessment of individual pain modulation profile (PMP).

A positive association between higher excitability of M1, efficient CPM/low TS magnitude, and analgesic responses would allow us to suggest that the neurophysiological and psychophysical domains of antinociception are interrelated. In this study, we aimed to test this hypothesis in a group of young healthy subjects, using a battery of tests for psychophysical and neurophysiological assessment. We hypothesized that higher corticospinal excitability would be associated with antinociceptive PMP.

## 2. Methods

Forty-one young healthy subjects (22 women) underwent comprehensive assessment of corticospinal excitability and pain psychophysics. In addition, all subjects filled a battery of pain-related psychological questionnaires. The questionnaire-related data will be reported separately. The inclusion criteria for the participants were as follows: age range of 18 to 40 years; no chronic or acute pain events; and no self-reported attention deficit. The subjects were asked to have a full night's sleep before the experimental session and to avoid caffeine consumption at least 2 hours before the experimental session. The Investigational Review Board of Rambam Health Care Campus, Haifa, Israel, approved the experimental protocol; the study protocol conformed to the ethical guidelines of the 1975 Declaration of Helsinki. Informed consent was obtained from all participants.

### 2.1. Neurophysiological measurements

We measured the resting motor threshold (rMT); the motor-evoked potential (MEP) latency, amplitude, and duration; and the duration of the cortical silent period (CSP) in the left right abductor pollicis brevis (APB) muscle in response to dominant M1 stimulation using a figure-of-eight coil (MCF-B65) with MagPro X100 magnetic stimulator (MagVenture, Inc., Farum, Denmark). The TMS preparations started with supporting the subject's neck using an airplane pillow and putting a tight swimmer's cap over the head to mark coil location and angulation. The exact coil orientation for APB stimulation was adjusted individually. Two surface electrodes in a tendon-belly construction were applied over the nondominant APB muscle for MEP recordings. Subjects were instructed to recline comfortably, keep eyes open, lean head back, and report any pain or discomfort on the head or muscle twitches in the hand. Participants were instructed to keep their hand relaxed throughout the experiment. We used the MEB-9400 electromyography (EMG)/evoked potentials (EP) system (Nihon Kohden, Tokyo, Japan) to record and analyze the waveforms with a bandpass filter at 5 Hz–10 kHz.

The measures of corticospinal excitability were defined as follows:

(1) Resting motor threshold was defined as the lowest stimulus intensity able to elicit MEPs at amplitudes of 50 μV in 5 of 10 consecutive stimuli when the muscle is at rest. The coil was placed above the contralateral M1 to the examined hand with the coil oriented at 45° towards the contralateral forehead. The coil was moved to determine the spot with maximal MEP amplitude.^58^ The TMS intensity was reported as the percentage of the maximal TMS machine output. Starting at 30% stimulus intensity, the intensity was increased incrementally until every stimulus resulted in a consistent MEP. Stimuli were given at interstimulus interval ±8 seconds to avoid facilitation.
(2) The MEPs were measured at 20% above the individual rMT in 8 to 10 successful (the response ≥50 μV) single trials. The MEPs were quantified as amplitude (mV) of the peak response, the onset of the MEP (MEP latency), and its duration from onset of the response to its return to the baseline (MEP duration). Motor-evoked potential duration was highly correlated with short-interval intracortical inhibition (SICI)^68^; therefore, we decided to use this measure, although it is not commonly used.
(3) The CSPs were measured in 6 to 8 single trials per stimulation intensity—at 100%, 120%, and 140% of the rMT, the stimuli were delivered while the subject performed a tonic voluntary contraction of nondominant APB muscle at 50% of maximal force. Data were subsequently averaged to provide one mean value per variable per measure or condition. The duration of the CSP was taken from the end of the MEP to the latency at which the EMG activity returned to its mean prestimulus level.

### 2.2. Psychophysics

(1) Heat pain thresholds were assessed by the method of limits.^74^ The TSA thermode was attached to the volar aspect of the nondominant forearm. Starting at a baseline temperature of 32°C, the thermode warmed at a rate of 1.5°C/s until pain sensation was perceived. This was repeated 3 times, and results were averaged to obtain a heat pain threshold value.
(2) Conditioned pain modulation was assessed using the parallel paradigm. The test-stimulus was a tonic noxious contact heat stimulus applied to the volar aspect of the dominant forearm using TSA. The intensity of the test-stimulus was predetermined individually based on the psychophysical parameter of Pain60 temperature.^23^ This method is based on delivery of several triplets of 7-second-long stimuli of various intensities; the closest temperature that induced pain at a level of 60 on a 0 (no pain) to 100 (the most imaginable pain) through the numerical pain score (NPS) was considered as the Pain60 temperature. For each stimulus, the baseline temperature was set at 32°C, which increased at a rate of 2°C/s to the destination temperature. The test-stimulus was applied for 30 seconds and decreased back to baseline at the same rate. Subjects rated the intensity of test-stimulus at 10th, 20th, and 30th second along stimulus duration. The mean pain score served as the pain level of “test-stimulus.” After a 15-minute break, subjects immersed their nondominant hand up to the wrist into a hot water bath at 46.5°C (Heto CBN 8-30 Lab equipment, Heto‐Holten A/S, Allerod, Denmark), for 1 minute (a conditioning stimulus). During the first 30 seconds of immersion, the conditioning stimulus was applied stand alone; subjects rated the pain intensity every 10 seconds; the mean score of the 3 pain ratings served the conditioning stimulus pain level. During the last 30 seconds of the conditioning stimulus, an identical test-stimulus was repeated, and pain of the test-stimulus was rated again every 10 seconds. The CPM effect was calculated as the difference between 2 test-stimuli: one applied under dual-stimulation vs the test-stimulus given stand alone. More negative values indicated more efficient CPM.^23,26,75,77^
(3) Electrical temporal summation (eTS) was measured by delivering electrical stimuli with a constant current stimulator (Digitimer DS5; Digitimer Ltd, WelWyn Garden City, England); 2 bipolar Ag/AgCl-electrodes were attached to the skin overlying the belly of the nondominant brachioradialis muscle. Square-wave 2-ms-long pulses were given. First, electrical pain threshold (ePT) was determined through the continued increase of stimulation intensity step of 1 mA, starting at 3 mA, until the participant indicates pain sensation. The eTS assessment was performed with stimulation intensity at 30% above the individual ePT. Ten repetitive stimuli were delivered with interstimulus interval of 1 second. The numerical pain score was obtained after first application and after the last of the ten stimuli. eTS magnitude was calculated as absolute difference between last and first pain scores. This stimulation protocol was recently published by our laboratory.^36^

### 2.3. Statistical analysis

Data were processed and analyzed by Excel (Microsoft Corp, Redmond, WA) and JMP (SAS Institute, Cary, NC) software.

Resting motor threshold; MEP amplitude, latency, and duration along with the CSP onset latency; and duration data were subjected to correlation analysis with the pain modulation measures (CPM and TS) and pain thresholds. No formal power or sample size analysis was performed before the study. A post hoc power analysis was performed to determine the minimum correlation, which would be regarded as significant based on the number of cases in the analysis, power of 0.80, and alpha = 0.05 Bonferroni-adjusted for 28 comparisons. The range of correlations is reported for the maximum and minimum number of cases actually included in correlation analyses.

All data are presented in mean ± SD unless indicated otherwise.

## 3. Results

### 3.1. The description of demographic characteristics, and psychophysical and neurophysiological responses

The mean age of the participants was 26.2 ± 4.2 years. Because we did not detect a significant correlation between subject age and any of psychophysical or neurophysiological variables, nor find any significant sex effect of any of the tested variables, we decided to exclude age and sex from further analyses. Post hoc analysis of the correlation values indicated that the largest analysis of 41 cases would find significance with *r* of at least ±0.57, and the analysis of 29 cases would find significance for *r* of at least ±0.66.

Thirty-nine of 41 subjects were right-handed.

The descriptive values for psychophysical and neurophysiological responses are presented in Table 1. Please note that the data on CPM or its counterparts (“test,” “conditioning,” and “conditioned” stimuli) are based on responses from 34 subjects, as 7 participants had a mean “test” pain score below 20 NPS. Based on previous experience of our and other laboratories, the pain scores <20 are considered as too mild a pain experience.^1,23,79^ Using this cutoff, we aimed to eliminate the possible floor effect on the test-stimulus pain scores.^29^

**Table 1:**
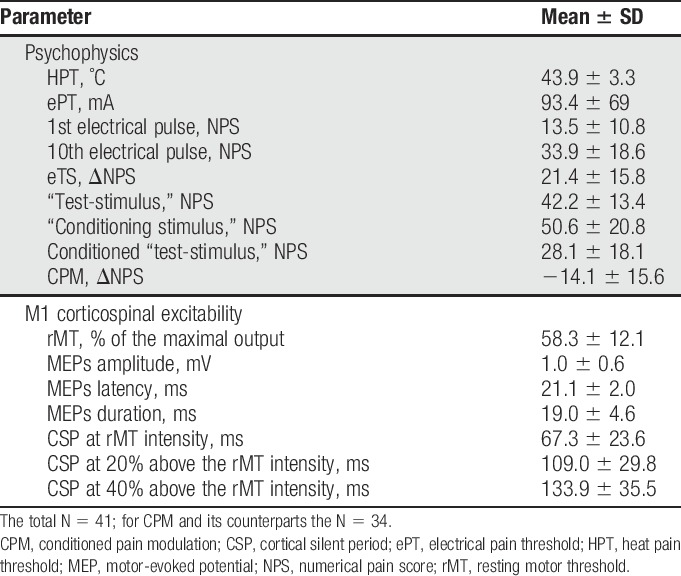
Psychophysical and neurophysiological characteristics of the study group.

### 3.2. The relationship between corticospinal excitability and psychophysics

**Figure 1.**
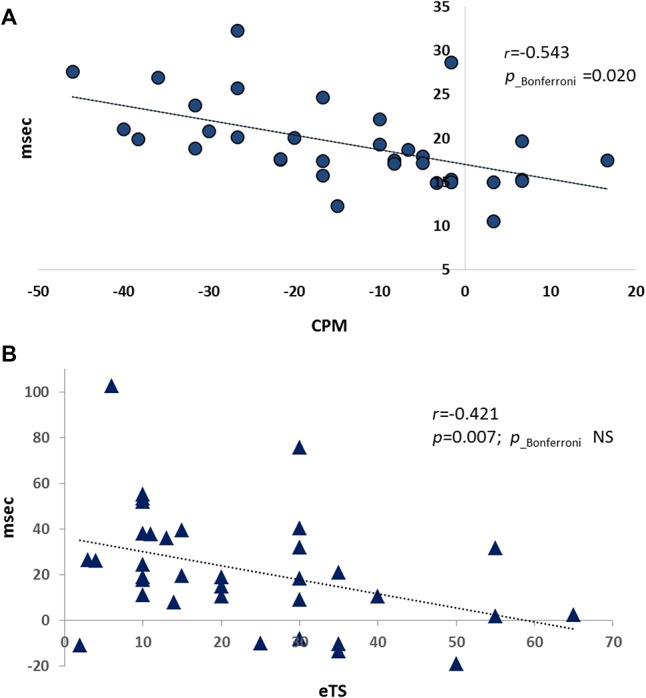
The MEP duration positively correlates with the CPM efficiency (A) and negatively correlates with the TS magnitude (B). CPM, conditioned pain modulation; MEP, motor-evoked potential; TS, temporal summation.

Among all psychophysical parameters, after correction for the multiply comparisons, the measures of corticospinal excitability significantly correlated with the extent of CPM (n = 34) (Table 2). More specifically, efficient CPM (negative values) was associated with higher MEP amplitudes and longer duration of MEPs. By contrast, high TS magnitude (positive values) was associated with shorter MEP duration; however, the statistical significance of this correlation was lost after multiplicity adjustment. The correlation plot of MEP duration and CPM/eTS is presented in Figure 1.

**Table 2:**
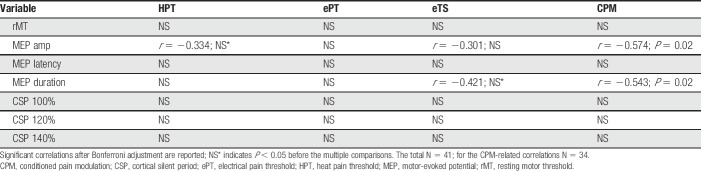
Correlations between psychophysical and M1 corticospinal excitability parameters.

## 4. Discussion

The results of our study demonstrate association between antinociceptive pattern of pain modulation as reflected by the correlations between efficient CPM and lower TS magnitude, on one hand, and higher excitability at the motor pathways, reflected by higher MEPs amplitude and its longer duration. This association was stronger for the CPM. This is the first study that tested the relationship between corticospinal excitability and parameters of pain modulation in healthy subjects. Our findings thus suggest that the MEP characteristics can serve a neurophysiological counterpart of the inhibitory facet of the individual PMP responses with a potential to be explored in clinical setups.

TMS can excite deep gray matter neurons either directly or indirectly through volleys from superficial neurons.^5^ Primary motor cortex stimulation evokes indirect excitation of pyramidal neurons through local interneurons with higher probability than direct excitation.^2^ These activation of the corticospinal pathway can elicit MEPs in the muscles contralateral to the muscle cortical representation in M1.^28^ Motor-evoked potentials are used routinely in research and under several clinical settings in evaluating motor cortex excitability. This evaluation should however differentiate between the indices of the overall corticospinal excitability and indices specific to the excitability of the motor cortex (cortical excitability). EMG in general and MEPs in particular are affected by a combination of cortical, subcortical, and spinal cord mechanisms, which usually coincide in time, making their separation almost impossible.

It is widely accepted that experimental pain has inhibitory influence on M1 excitability in healthy subjects. Most studies reported reduced MEP amplitudes in response to various pain modalities such as capsaicin cream application,^12^ noxious heat,^19,44^ or acute muscle pain.^9,38^ In line, activation of the motor cortex induced by physical exercise or rTMS/cathodal (inhibitory) transcranial direct current stimulation (tDCS) has inhibitory effect on experimental pain as reflected by increased pain thresholds^48,49^ and reduced pain perception.^20,25^ Thus, we can relate the higher level of corticospinal excitability in healthy state to one of the neurophysiological correlates of antinociception.

In chronic pain states, enhanced M1 excitability loses its pain inhibitory functions, probably due to the pain-related cortical reorganization.^59^ M1 excitability in chronic states and syndromes such as neuropathic pain, myofascial pain syndrome (MPS), and fibromyalgia is characterized by reduced CSP, lower SICI, and enhanced intracortical facilitation (ICF) and MEPs.^10,11,54,60–62,66,70^ In contrast to the normal state, M1 excitability in pain patients is inversely correlated with inhibitory pain modulation as was demonstrated by higher MEPs observed in those patients with MPS who failed to induce efficient CPM response.^7^ Increased MEPs in MPS were also observed after exposure to experimental pain further pointing to the disinhibition of corticospinal system.^70^ Furthermore, the pain-relieving effect of M1-directed rTMS treatment is often parallel with activation of inhibitory mechanisms of homeostatic plasticity (normalization) of the motor cortex excitability.^8,14,40,41^ Importantly, high-frequency rTMS has different impact on the motor cortex excitability in healthy subjects vs chronic pain patients, probably due to self-limiting hyperexcitability capacity. It increases MEPs in controls but decreased it in migraine patients with aura,^8^ prolongs SICI in neuropathic pain,^40,41^ reduces ICF, and enhances MEPs in patients with MPS.^15^ Harmonizing with abnormal M1 excitability, many chronic pain syndromes are characterized by less-efficient CPM and/or high TS and therefore can be anchored to the pronociceptive edge of the nociception spectrum.^42,52,78^ We believe therefore that the strong correlation between MEPs and CPM efficiency reported in our study may carefully suggest the additive value of motor corticospinal excitability for comprehensive assessment of individual PMP.

The neurophysiological basis of our findings requires explanation. Beside the expected correlation with the amplitude, CPM efficiency was significantly associated with MEP duration. There are a number of physiological processes which are likely to be involved in the increase in MEP duration, and they are likely to operate at different levels of the neuraxis. Single TMS pulse applied to M1 gives rise to a series of descending corticospinal volleys.^18,27^ The preferred diagnostic coil is circular; however, we used a figure-of-eight coil, which is more focal and demands exact coil placement over the stimulated motor cortex area of the specific muscle,^27^ as was performed in this study. At spinal level, these volleys activate motoneurons at slightly different latencies, dependent on their thresholds. This asynchronization results in MEPs with prolonged duration and lower amplitude.^57^ In case of higher neural excitability at the cortical or spinal level, more motor neurons will exceed the threshold resulting in enhanced MEP amplitudes and longer duration, without a change in stimulus intensity. This implies that the stimulus–response curve, reflecting the relation between stimulus intensity and MEP amplitude and duration, is subject to dynamic changes that relate to the present physiological state of the motor system.^56,64^ Furthermore, there are reports of a significant inverse relationship between SICI and facilitated MEP duration, suggesting a contribution of cortical processes in prolongation of facilitated MEP duration.^68^ Correspondingly, the lack of correlation between SICI and the facilitated MEP amplitude is consistent with existing evidence that facilitation of MEP amplitude during a tonic voluntary contraction is primarily spinal.^17,30^ It is therefore possible that longer MEPs may represent more indirect volleys contribution to the final MEP, which, in turn, could constitute a marker of more effective intrinsic pain inhibitory top-down mechanisms.

On the biochemical level, MEPs represent the net facilitatory effect of a TMS pulses that engage the excitation of the motor cortex mediated by 4 major neuromodulatory neurotransmitter systems; glutamate, acetylcholine, dopamine, and noradrenaline.^32,34,35,39,47^ We may hypothesize therefore that higher MEPs reflect higher level of corticocortical and corticospinal glutamatergic, cholinergic, dopaminergic, and noradrenergic neurotransmission. In line, the activity of these neuropharmacological systems in the motor cortex may be involved in its connectivity with cortical and subcortical areas associated with pain processing and inhibitory pain modulation. Indeed, the mechanism of the rTMS-evoked analgesia implies rapid and phasic activation in the lateral thalamus, which leads to a cascade of synaptic events influencing activity in the medial thalamus and in the brain structures involved in descending pain inhibition such as perigenual anterior cingulate cortex, orbitofrontal cortex, and periaqueductal gray.^21,22,46^ In addition to the established involvement of opioid neurotransmission, cellular mechanisms of the rTMS-M1 analgesic effect, at least partially, are mediated by the glutamate^13^ and may involve other neurotransmitters^46^ Thus, we can assume that intracortical neuropharmacological systems might indirectly contribute to the efficiency of antinociception.

Among the parameters of the motor corticospinal excitability applied in this study, we explored the CSP—the arrest of motor cortex activity due to single suprathreshold TMS pulse given during muscle contraction. Its length is believed to reflect the activity of inhibitory interneurons,^14^ mediated by GABA-B receptors^72^ and cholinergic neurotransmission^31,32^; its shortening indicates deficient GABA-B–mediated intracortical inhibition reported also in pain patients. Our results did not reveal substantial relationship between the CSP prolongation at any stimulation intensity, and for any pain psychophysics parameter. This may indicate lower relevance of motor corticospinal GABAergic transmission to processing of the experimental pain.

Our study has several limitations. The main limitation is the restriction of evaluation of motor corticospinal excitability. This restricted assessment of motor cortex excitability is related to the fact that the TMS system we used could not perform a paired-pulse stimulation required for the assessment of SICI and ICF—the most commonly applied methods for the neurophysiological assessment of primary motor cortex. The main findings of our study refer to the measures of MEPs; however, beyond intracortical processes, this response magnitude depends also on the corticospinal transmission and, therefore, could be affected by individual functioning of the motor system. We believe that this factor has minimal or no influence on our results, as our participants were young healthy subjects with no suspect of any neurological disease. Another study limitation is that the researcher who performed offline excitability data extraction was not blinded to the results of psychophysical assessment.

To conclude, the results of our study demonstrated a relationship between the neurophysiological and psychophysical tests of pain modulation; higher motor corticospinal excitability as reflected by larger MEP amplitudes and duration was associated with antinociceptive pattern of pain modulation. The described relationship may advance the development of comprehensive neurophysiology-based measures for pain modulation. We believe that objectification of pain modulation assessment will contribute to its use in the clinical setting.

## Disclosures

The authors have no conflict of interest to declare.
